# Syphilology and Venereal Disease

**Published:** 1907-03

**Authors:** 


**Syphilology and Venereal Disease.** By C. F. MARSHALL, M.D., M.Sc., F.R.C.S. Pp. x., 509. London: Baillière, Tindall and Cox. 1906.

Dr. Marshall's book on syphilis is one of the most valuable monographs that has appeared of recent years. Inspired avowedly by the discovery of the *spirochæta pallida* by Schaudinn and Hoffmann in 1905, and accepting under reserve their claim for this as the specific organism of the disease, yet so complete is the study of syphilis in all its bearings, that were the part played by the spirochæta proved to be something less than at present appears likely, still Marshall's work must remain a very valuable landmark in the field of scientific literature. The inclusion of a complete bibliography of classical and modern authorities stamps the book as indispensable to the scientific investigator, while the concise and exhaustive description of syphilis from every standpoint is set out with such impartial judgment and wealth of detail that no practitioner can afford to leave the book unread.

The subject is one that intrudes itself into every malady; and the effects of syphilis, its relationships, whether clinical, psychological or sociological, in the individual or the species are of such overwhelming moment, that so helpful a contribution to our knowledge deserves from the critic nothing but commendation.

Although the modern developments of science are fully dealt with, the style in which they are expounded does not require any very extraordinary pathological knowledge to be appreciated by the reader: even one who is not in close touch with modern laboratory methods will find himself informed, not puzzled, by Dr. Marshall's chapters in this province.

The experimental work upon *inoculation* both in man and monkeys is adequately described, and throws fresh light on the infectivity of the disease, not only as regards the length of time during which the acquired form may remain communicable, but also as to the transmission of the inherited taint.

Marshall summarises the evidence in favour of the spirochæta pallida as the specific organism of syphilis thus:—

"1. It has been found almost constantly in the primary and secondary lesions whether ulcerated or not.

"2. It has also been found in the blood.

"3. The same parasite has been found in widely-separated countries.

"4. It has been found in the blood and viscera of the syphilitic fœtus.

"5. It has been found by Metchnikoff and Roux in the syphilitic lesions of monkeys inoculated both with human virus and virus from other syphilitic monkeys.

"6. It has not been found in man or monkeys apart from syphilitic lesions."

The only one of Koch's postulates not complied with so far is that the spirochæta pallida has not yet been cultivated out of the body. But the other evidence certainly seems incontrovertible that this is actually the specific cause of syphilis.

Unfortunately, although this conclusion may be accepted, all efforts hitherto made to discover a method of preventing the infection by immunisation or treatment have been in vain.

Even hereditary syphilis has proved no protection against the acquisition of the disease in later life by the ordinary means of infection.

The *clinical manifestations* and *treatment* are described with a fulness to which we can only refer with admiration, in the hope that a closer study of the recent improvements in treatment may lead to their wider adoption and the ultimate removal of the reproach under which the medical profession at present (perhaps justly) lies, that in England syphilis is frequently untreated and always inadequately.

The so-called *parasyphilitic lesions*—that is, diseases which owe their origin to, but do not depend upon any pathological change characteristic of syphilis, occupy a very important place in the book on account of the valuable researches of Mott, Ferrier, and others.

*Hereditary syphilis* and the proved possibility of transmission to the third generation form perhaps, together with the advances in pathology, the most interesting reading that Dr. Marshall affords. The summary of facts observed in connection with the inheritance of the disease is well worth quoting:—

"1. That the degenerative effects of syphilis are frequently transmitted to the third generation, and possibly further, only to die out with eventual sterility.

"2. That, although difficult to prove, the transmission of virulent hereditary syphilis to the next generation is possible, and depends chiefly on two factors, *time and treatment*. . . . The reason why such cases are not more common lies in the fact that comparatively few subjects marry while suffering from hereditary syphilis in an active state.

"3. That re-infection of a heredo-syphilitic genitor increases the virulence of the disease and its fatal effects on the offspring.

"4. That the two chief obstacles to actual proof of transmission to the third generation are the possible re-infection of the second generation and the possible intervention of another syphilitic genitor. . . . For although a child is obviously the offspring of its mother, paternal parentage is seldom capable of scientific proof.

"5. That the hereditary transmission of syphilis is one of the chief factors in physical, mental and moral degeneration."

Hereditary syphilis is insisted upon as essentially contagious, and to belittle this fact is a dangerous doctrine.

Syphilis is described as the hereditary disease *par excellence*, its hereditary effects are more inevitable, more multiple, more diverse and more disastrous in their results on the progeny and the race than is the case in any other disease. It has, in fact, a more harmful influence on the species than on the individual. Dr. Marshall acknowledges his indebtedness to the teaching of Fournier and his disciples; we in turn must with no less gratitude confess to the debt which we owe to the English exponent of their doctrines.

